# Cluster radioactivity of neutron-deficient nuclei in trans-tin region

**DOI:** 10.1038/s41598-020-65585-x

**Published:** 2020-06-04

**Authors:** Yonghao Gao, Jianpo Cui, Yanzhao Wang, Jianzhong Gu

**Affiliations:** 1 Department of Mathematics and Physics, Shijiazhuang Tiedao University, Shijiazhuang, 050043 China; 2 Institute of Applied Physics, Shijiazhuang Tiedao University, Shijiazhuang, 050043 China; 3 Department of Physics, Royal Institute of Technology (KTH), SE-10691 Stockholm, Sweden; 4 China Institute of Atomic Energy, P. O. Box 275 (10), Beijing, 102413 China

**Keywords:** Experimental nuclear physics, Theoretical nuclear physics

## Abstract

The possibility of cluster radioactivity (CR) of the neutron-deficient nuclei in the trans-tin region is explored by using the effective liquid drop model (ELDM), generalized liquid drop model (GLDM), and several sets of analytic formulas. It is found that the minimal half-lives are at *N*_*d*_ = 50 (*N*_*d*_ is the neutron number of the daughter nucleus) for the same kind cluster emission because of the *Q* value (released energy) shell effect at *N*_*d*_ = 50. Meanwhile, it is shown that the half-lives of *α*-like (*A*_*e*_ = 4*n*, *Z*_*e*_ = *N*_*e*_. *Z*_*e*_ and *N*_*e*_ are the charge number and neutron number of the emitted cluster, respectively.) cluster emissions leading to the isotopes with *Z*_*d*_ = 50 (*Z*_*d*_ is the proton number of the daughter nucleus) are easier to measure than those of non-*α*-like (*A*_*e*_ = 4*n* + 2) cases due to the large Q values in α-like cluster emission processes. Finally, some *α*-like CR half-lives of the *N*_*d*_ = 50 nuclei and their neighbours are predicted, which are useful for searching for the new CR in future experiments.

## Introduction

In recent years, the CR of unstable heavy nuclei has received attention by many researchers^1–26^. The CR was first predicted in 1980 by Sandulescu, Poenaru and Greiner^27^, and then it was confirmed by Rose and Jones in 1984 for the ^14^C radioactivity from ^223^Ra^28^. From then on, the emissions of ^14^C, ^20^O, ^23^F, ^22,24−26^Ne, ^28,30^Mg and ^32,34^Si, have been experimentally observed in the mass region where the parent nuclei with their charge numbers *Z* = 87–96^29–33^. In this region all cluster emissions have closed shell daughters, *i.e*. the daughter nuclei are ^208^Pb or its neighbors. It is well known that *α*-decay is an important decay mode for unstable heavy nuclei^34–37^, which can be described by the quantum tunneling effect through a potential barrier^38–56^. Usually the CR is seen as a cold asymmetric fission process, whose case is similar to *α*-decay. On the basis of the fission knowledge^57,58^ and the quantum tunneling effect, many phenomenological and microscopic models were developed to construct the potential barrier of CR, and furthermore to estimate the half-life^1–26^. In addition to these models, many analytic formulas were proposed by fitting the experimental half-lives and *Q* values of CR processes, such as the UDL^59,60^, UNIV^61^, Horoi^62^, TM^63^, BKAG^64^, NRDX^65^, and VSS^66^ formulas.

Besides the CR of the parent nuclei with *Z* = 87–96, two new islands of cluster emitters have been predicted by many models^67–90^. One is in the superheavy nuclei (SHN) region^67–75^, the other is the in the trans-tin region decaying into the daughter nuclei close to ^100^Sn^76–90^. For the CR of the SHN, Poenaru *et al*. changed the concept of the CR to allow emitted particles with *Z*_*e*_ > 28 from the parents with *Z* > 110 (daughter around ^208^Pb). They found that the CR is one of the most important decay modes and its branching ratio is larger than that of the *α*-decay for *Z* ≥ 121 nuclei by the analytic superasymmetric fission (ASAF) model^69–71^. Additionally, it is shown that the shell effects at ^208^Pb and *N* = 184 strongly influence the CR half-lives^69–71^. Later, the calculations within several models gave similar predictions to that of the ASAF model^72,73^. For the CR in the trans-tin region, the half-life of the ^12^C emission of ^114^Ba has been measured by Oganessian *et al*. at Dubna (Dubna94)^91^ and by Guglielmetti *et al*. at GSI (GSI95)^92,93^. The obtained experimental half-lives of Dubna94 and GSI95 were ≥10^3^ s^91^ and ≥1.1 × 10^3^ s (1.7 × 10^4^ s)^92,93^, respectively. However, the ^12^C decay of ^114^Ba was not observed in the later measurement of Guglielmetti *et al*.(GSI97)^94^, which suggested the branching ratio for the ^12^C decay is lower than the limit obtained in the GSI95 experiment. By consulting the NUBASE2016 Table the experimental lower limit of the half-life of the ^12^C emission from ^114^Ba is found to be >4.13 s (in logarithmic scale)^95^. So the half-life of the ^12^C radioactivity from ^114^Ba has not yet been determined accurately.

As a matter of fact, the CR of the trans-tin region has been predicted since 1989^96^. In recent decades, the CR half-lives of the emitters from ^8^Be to ^32^S have been estimated within many models by inputting different kinds of *Q* values^76–90^. Very recently, the CR of the SHN was studied systematically by several models. It is shown that the CR half-lives are strongly dependent on the models used^97^. This drives us to wonder that in the trans-tin region whether the CR island exists if other models are employed. Furthermore, whether the CR half-lives extracted from different models are similar to each other if we input the same *Q* values. This constitutes the motivation of this article. In this article, we will explore the CR of neutron-deficient nuclei in the trans-tin region and examine the model dependence of half-lives using the ELDM, GLDM, and several sets of analytic formulas (UDL, UNIV, Horoi, TM, BKAG, NRDX and VSS formulas). The paper is organized as follows. In section 2, the theoretical approaches are introduced. The numerical results and discussions are presented in section 3. Some conclusions are drawn in the last section.

## Models

The ELDM and GLDM are successful models for describing the processes of proton emission, *α*-decay, and CR in a unified framework. The details of them can be found in refs. ^10–16^.

In the unified fission model the partial half-life of a cluster emitter is simply defined as

1 $$T=\frac{\mathrm{ln}\,2}{{\nu }_{0}P},$$

where *ν*_0_ is the frequency of assaults on the barrier. *P* is the barrier penetration probability.

For the ELDM, in the combination of the Varying Mass Asymmetry Shape and Werner-Wheeler’s inertia, the *ν*_0_ value is taken as 1.0 × 10^22^ s^−1^ ^10–13^, and *P* is calculated by

2 $$P=\exp \left[-\frac{2}{\hslash }{\int }_{{\zeta }_{0}}^{{\zeta }_{c}}\sqrt{2\mu [V(\zeta )-Q]}d\zeta \right],$$

where *μ* is the Werner-Wheeler’s inertia inertial coefficient. *ζ*_0_ and *ζ*_*c*_ are the inner and outer classical turning points, respectively. The two classical turning points are expressed as ${\zeta }_{0}=R-{\bar{R}}_{1}$ and *ζ*_*c*_ = $\frac{{Z}_{e}{Z}_{d}{e}^{2}}{2Q}+\sqrt{{\left(\frac{{Z}_{e}{Z}_{d}{e}^{2}}{2Q}\right)}^{2}+\frac{l(l+1){\hslash }^{2}}{2\mu Q}}$, respectively. Here *R* is the radius of the parent nucleus. ${\bar{R}}_{1}$ represents the the final radius of the emitted cluster.

The effective one-dimensional total potential energy is given by^10–13^

3 $$V={V}_{c}+{V}_{s}+{V}_{l}\mathrm{}.$$

The Coulomb contribution *V*_*c*_ is determined by using an analytical solution of the Poisson’s equation for a uniform charge distribution system. The effective surface potential can be calculated by

4 $${V}_{s}={\sigma }_{eff}({S}_{e}+{S}_{d}),$$

where *S*_*e*_ and *S*_*d*_ are the surface areas of the two spherical fragments. *σ*_*eff*_ is the effective surface tension, which is defined as

5 $${\sigma }_{eff}=\frac{1}{\mathrm{4(}{R}^{2}-{\bar{R}}_{1}^{2}-{\bar{R}}_{2}^{2})}\left[Q-\frac{3}{20\pi {\varepsilon }_{0}}{e}^{2}\left(\frac{{Z}^{2}}{R}-\frac{{Z}_{e}^{2}}{{\bar{R}}_{1}}-\frac{{Z}_{d}^{2}}{{\bar{R}}_{2}}\right)\right],$$

where ${\bar{R}}_{2}$ is the final radius of the daughter fragment.

The centrifugal potential energy beyond the scission point has an usual expression

6 $${V}_{l}=\frac{{\hslash }^{2}}{2\bar{\mu }}\frac{l(l+\mathrm{1)}}{{\zeta }^{2}},$$

where *l* is the angular momentum of the emitted particle, $\bar{\mu }={M}_{e}{M}_{d}/({M}_{e}+{M}_{d})$ is the reduced mass of the two separated fragments. *M*_*e*_ and *M*_*d*_ represent their atomic masses.

In the framework of the GLDM, *ν*_0_ is givn by the following classic method^14–16^

7 $${\nu }_{0}=\frac{1}{2R}\sqrt{\frac{2{E}_{e}}{{M}_{e}}},$$

where *E*_*e*_ and *M*_*e*_ are the kinetic energy and mass of cluster, respectively.

*P* is calculated by using the WKB approximation, which is written by

8 $$P=\exp \left[-\frac{2}{\hslash }{\int }_{{R}_{{\rm{in}}}}^{{R}_{{\rm{out}}}}\sqrt{2B(r)(E(r)-{E}_{sph})}dr\right]\mathrm{}.$$

The deformation energy (relative to the sphere) is small up to the rupture point between the fragments. *R*_in_ is the distance between the mass centers of the portions of the initial sphere separated by a plane perpendicular to the deformation axis to assume the volume conservation of the future fragments. *R*_*out*_ = $\frac{{Z}_{e}{Z}_{d}{e}^{2}}{2Q}+\sqrt{{\left(\frac{{Z}_{e}{Z}_{d}{e}^{2}}{2Q}\right)}^{2}+\frac{l(l+1){\hslash }^{2}}{2\mu Q}}$. The inertia *B*(*r*) = *μ*(1 + 1.3 *f*(*r*)), which can simulate a rapid variation of the friction force effects only at the moment of the neck rupture between the nascent fragments. If *r* ≤ *R*_*cont*_, $f(r)=\sqrt{\frac{{R}_{cont}-r}{{R}_{cont}-{R}_{in}}}$. Otherwise, *f*(*r*) = 0^14–16^. Here *R*_*cont*_ = *R*_*e*_ + *R*_*d*_, *R*_*e*_ and *R*_*d*_ are the radii of the cluster and daughter nucleus, respectively.

The analytic formulas (UDL^59,60^, UNIV^61^, Horoi^62^, TM^63^, BKAG^64^, NRDX^65^, and VSS^66^ formulas) used in this article are expressed as

9 $${\log }_{10}{T}_{\mathrm{1/2}}({\rm{UDL}})=a\sqrt{\mu }{Z}_{e}{Z}_{d}{Q}^{-\mathrm{1/2}}+b{[\mu {Z}_{e}{Z}_{d}({A}_{e}^{\mathrm{1/3}}+{A}_{d}^{\mathrm{1/3}})]}^{\mathrm{1/2}}+c,$$

10 $$\begin{array}{rcl}{\log }_{10}{T}_{\mathrm{1/2}}({\rm{UNIV}}) & = & a{(\mu {Z}_{e}{Z}_{d}{R}_{b})}^{\mathrm{1/2}}\times [\arccos \sqrt{r}-\sqrt{r\mathrm{(1}-r)}]\\  &  & +b({A}_{e}-\mathrm{1)}+[{\log }_{10}(\mathrm{ln}\,\mathrm{2)}-{\log }_{10}{\nu }_{0}],\end{array}$$

11 $${\log }_{10}{T}_{\mathrm{1/2}}({\rm{Horoi}})=(a{\mu }^{0.416}+b[({Z}_{e}{Z}_{d}{)}^{0.613}{Q}^{-\mathrm{1/2}}-\mathrm{7]}+(c{\mu }^{x}+d),$$

12 $$\begin{array}{l}{\log }_{10}{T}_{\mathrm{1/2}}({\rm{TM}})=(a{Z}_{e}+b)({Z}_{d}/Q{)}^{\mathrm{1/2}}+c{Z}_{e}+d,\end{array}$$

13 $$\begin{array}{l}{log}_{10}{T}_{\mathrm{1/2}}({\rm{BKAG}})=(a{A}_{e}\eta +b{Z}_{e}{\eta }_{z}){Q}^{-\mathrm{1/2}}+c,\end{array}$$

14 $$\begin{array}{l}{\log }_{10}{T}_{\mathrm{1/2}}({\rm{NRDX}})=a\sqrt{\mu }{Z}_{e}{Z}_{d}{Q}^{-\mathrm{1/2}}+b\sqrt{\mu }{({Z}_{e}{Z}_{d})}^{\mathrm{1/2}}+c,\end{array}$$

15 $$\begin{array}{l}{\log }_{10}{T}_{\mathrm{1/2}}({\rm{VSS}})=a{Z}_{e}{Z}_{d}{Q}^{-\mathrm{1/2}}+b{Z}_{e}{Z}_{d}+c+d,\end{array}$$

where *T*_1/2_ is the CR half-life, which is measured in seconds. *μ* = *A*_*e*_*A*_*d*_/(*A*_*e*_ + *A*_*d*_) is the reduced mass. *A*_*e*_ and *A*_*d*_ represent the mass numbers of the emitted particle and daughter nucleus, respectively. *Z*_*e*_ and *Z*_*d*_ denote the charge numbers of the two fragments. In Eq. (10), *r* = *R*_*t*_/*R*_*b*_, *R*_*t*_ and *R*_*b*_ stand for the first and second turning points of the barrier, respectively. The two turning points are defined as *R*_*t*_ = 1.2249(${A}_{e}^{1/3}$ + ${A}_{d}^{1/3}$) and *R*_*b*_ = 1.43998*Z*_*e*_*Z*_*d*_/*Q*. The frequency of assaults *ν*_0_ is taken as 10^22.01^ s^−1^. In Eq. (13), *η* (*η*_*z*_) represents the mass (charge) asymmetry, whose form is written as $\eta =\frac{{A}_{d}-{A}_{e}}{A}$ $({\eta }_{z}=\frac{{Z}_{d}-{Z}_{e}}{Z})$. The parameters in Eqs. (9–15) are determined by fitting the experimental half-lives and *Q* values^60–66^, which are listed in Table 1.

**Table 1:** The parameter sets of UDL, UNIV, Horoi, TM, BKAG, NRDX, and VSS formulas.

Parameters	UDL^60^	UNIV^61^	Horoi^62^	TM^63^	BKAG^64^	NRDX^65^	VSS^66^
*a*	0.3949	0.22873	9.1	12.8717	10.603	0.3998	1.51799
*b*	−0.3693	0.598	−10.2	−5.1222	78.027	−1.13263	−0.053387
*c*	−23.7615	—	7.39	−4.6496	−80.669	−21.85863	−92.91142
*d*	—	—	−23.2	−73.3326		—	1.402

## Results and discussions

It is well known that the CR half-lives are dependent on the *Q* values, which can be extracted by

16 $$Q=M-({M}_{d}+{M}_{e}),$$

where *M*, *M*_*d*_ and *M*_*e*_ represent the masses of the parent nucleus, daughter nucleus and emitted particle, respectively. The experimental nuclear masses are taken from ref. ^95^. For the unknown nuclear masses, in the CR half-life calculations whose values can be replaced by the theoretical nuclear masses extracted from the WS4 mass model^98^ because relevant studies showed that the WS4 mass model can predict the experimental nuclear masses and decay energies accurately^98,99^. Especially for our recent work on SHN, it suggested that the WS4 mass model is the most accurate one to reproduce the experimental *α*-decay energies of the SHN^100^.

Firstly, we calculate the ^12^C decay half-life of ^114^Ba using the ELDM, GLDM and some analytic formulas (UDL, UNIV, Horoi, TM, BKAG, NRDX and VSS formulas) and further test the predicted accuracies of these models by comparing to the experimental half-life. The calculated and experimental half-lives are presented in Table 2. The first and second columns are the parent nucleus and daughter nucleus, respectively. The released energy *Q* is listed in column 3^101^. Columns 4–12 give the ^12^C decay half-lives of ^114^Ba extracted from all the models and formulas. The last column lists the experimental half-life of the ^12^C decay from ^114^Ba^94^. According to Table 2, one can see that only the calculated half-lives by the NRDX and VSS formulas are below the experimental lower limit. The two formulas are simple scaling laws and the coefficients are determined by fitting the experimental data with the parent charge number *Z* = 87–96^65,66^. When they are extended to calculate the CR half-lives in trans-tin region, the predicted half-lives deviate from the experimental data. This indicates that the two scaling laws are not so universal and not suitable for estimating the CR half-lives in the trans-tin region. So, the two formulas will not be used to predict the CR half-lives in later calculations. In the following paragraphs by taking ^12^C, ^20^Ne and ^28^Si emissions as examples, the CR half-lives will be predicted by all the models (formulas) except for the NRDX and VSS formulas.

**Table 2:** Comparison between the experimental half-life of the ^12^C radioactivity of ^114^Ba and the estimated ones by the ELDM, GLDM and 7 formulas (The UDL, UNIV, Horoi, TM, BKAG, NRDX and VSS formulas).

Parent nucleus	Daughter nucleus	*Q*^*Expt*.^(MeV)	log_10_*T*_1/2_ (s)									
			ELDM	GLDM	UDL	UNIV	Horoi	TM	BKAG	NRDX	VSS	Expt.
^114^Ba	^102^Sn	19.00^101^	10.78	9.90	9.99	10.87	4.75	15.74	26.76	4.02	−4.40	>4.10^94^

The experimental half-life and *Q* value are taken from ref. ^94^ and ref. ^101^, respectively. The *Q* value and half-lives are measured in MeV and seconds, respectively.

**Figure 1:**
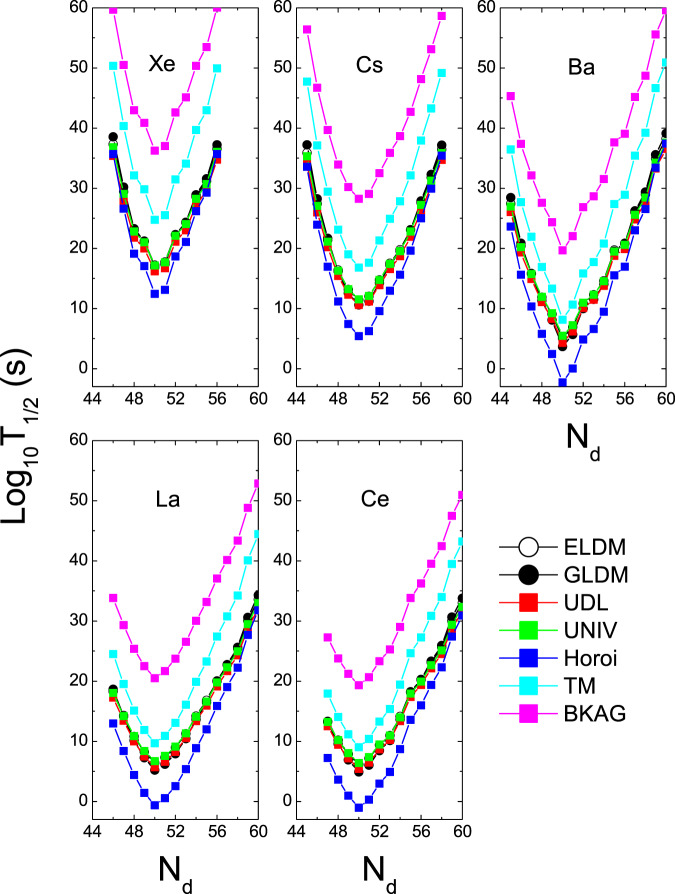
The ^12^C decay half-lives of the Xe, Cs, Ba, La, and Ce isotopes within the ELDM, GLDM, UDL, UNIV, Horoi, TM, and BKAG models (formulas) versus the neutron numbers of the daughter nuclei *N*_*d*_.

**Figure 2:**
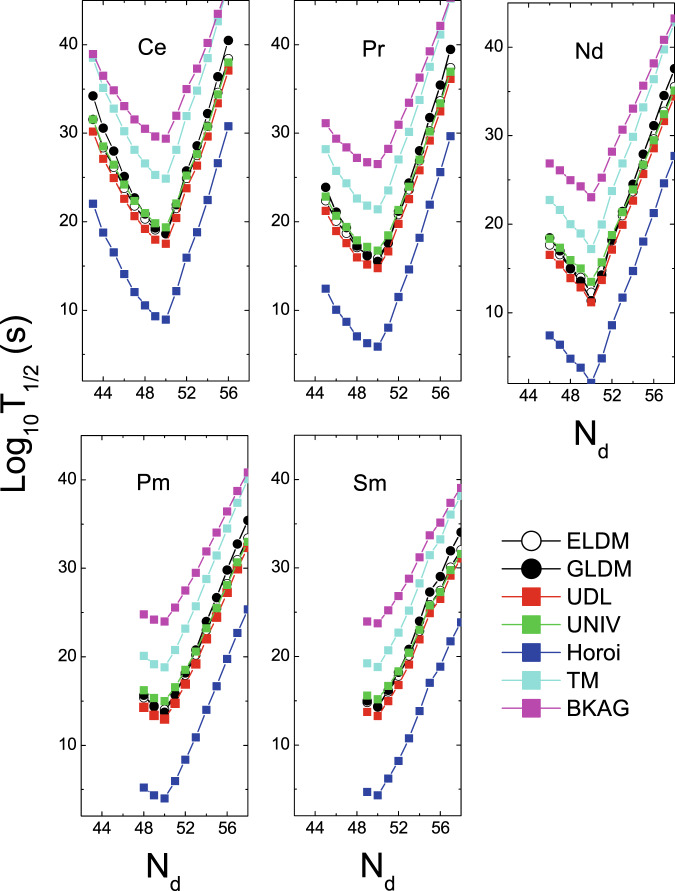
Same as Fig. 1, but for the ^20^Ne decay half-lives in the Ce, Pr, Nd, Pm, and Sm isotopes.

**Figure 3:**
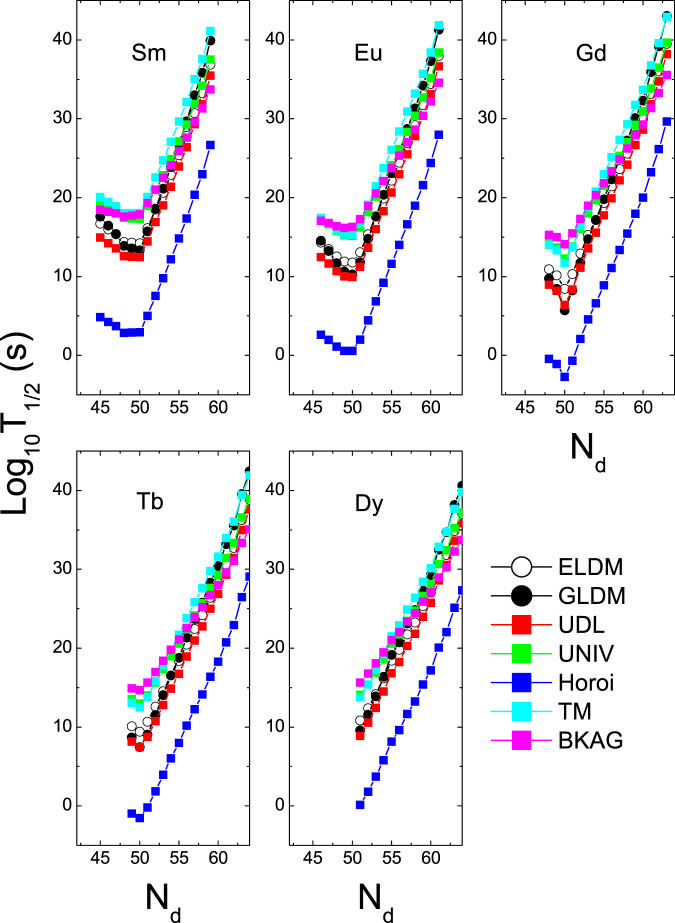
Same as Figs. 1 and 2, but for the ^28^Si decay half-lives in the Sm, Eu, Gd, Tb, and Dy isotopes.

**Figure 4:**
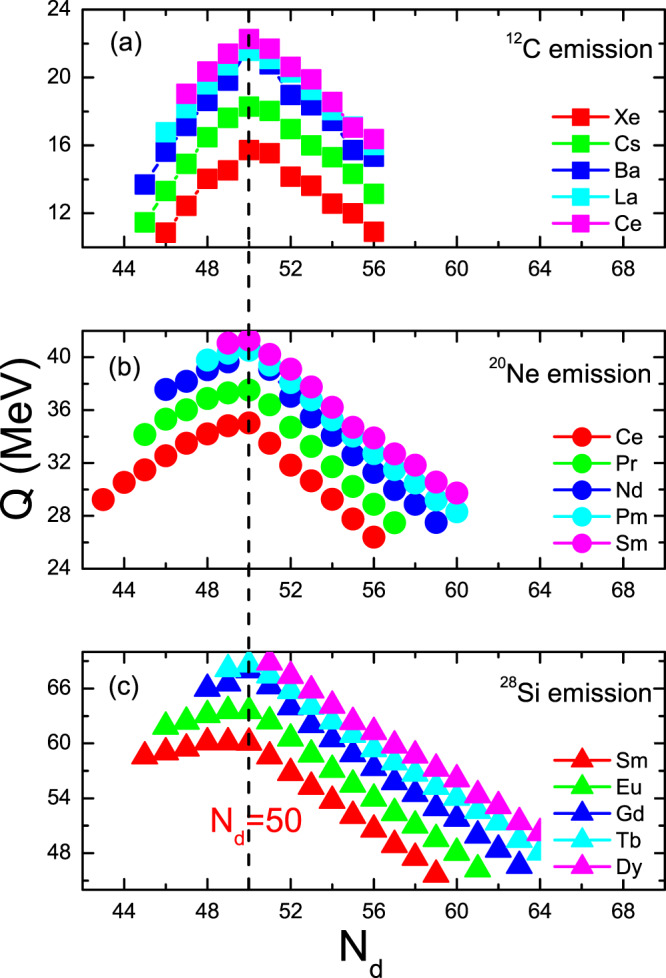
The *Q* values of the ^12^C, ^20^Ne, and ^28^Si emissions in some isotopic chains versus *N*_*d*_.

The half-lives of the ^12^C, ^20^Ne and ^28^Si emissions of some isotopes within the ELDM, GLDM, UDL, UNIV, Horoi, TM, and BKAG models (formulas) as functions of the daughter neutron number *N*_*d*_ are plotted in Figs. 1–3. Note that in the calculations by the ELDM and GLDM, the angular momenta carried by emitted particles are selected as 0. From Figs. 1–3, we can see that for each isotopic chain the CR half-lives calculated by the ELDM, GLDM, UDL and UNIV are almost the same. In the ELDM and GLDM, the cluster decay process is assumed as a super-asymmetric fission. The shape evolution process from one spherical nucleus to two separated fragments can be described well by the two models^10–16^. The shape evolution described by the two models contains more important nuclear structure information. In the ELDM the contributions of the Coulomb and surface energies to the potential barrier are considered more reasonably. The Coulomb energy is obtained by the exact solution of the Poisson’s equation for the system with a uniform charge distribution. For the surface potential energy, an effective surface tension is introduced. In addition, the inertial coefficient in the prescission phase is calculated with the Werner-Wheeler’s approximation^10–13^. In the GLDM, with the quasimolecular shape sequence and nuclear proximity energy, a reasonable configuration of the potential barrier can be obtained. Besides these factors, the accurate nuclear radius, decay asymmetry and assumed decay path are used as well. Thus, the charged particle emissions and nuclear fission can be described successfully by the two models^14–16^. Due to these advantages of the ELDM and GLDM, the predicted half-lives by them for yet unmeasured cluster emissions are more reliable than those by other phenomenological models. So to some extent the ELDM and GLDM can be seen as the standard models for estimating the half-lives of cluster emissions. As to the UDL and UNIV formulas, they are derived from from the *α*-like *R*-matrix theory and the fission-like theory, respectively^59–61^. Reasonable physical bases are behind them so that the CR half-lives extracted from the ELDM and GLDM are reproduced with a comparable accuracy by both of the formulas. Here it is worth mentioning that the experimental *α*-decay half-lives of SHN can be reproduced well by the UNIV formula^100^. But for the half-lives given by the Horoi^62^, TM^63^, and BKAG^64^ formulas, it is seen from Fig. 1 that they deviate from those by the ELDM and GLDM. Because the three formulas are the simple scaling laws^62–64^, which are similar to the NRDX and VSS formulas^65,66^. Although a little nuclear structure information is taken into account, their prediction power is not so strong. Moreover, from Fig. 1 the shortest half-lives appear when *N*_*d*_ is 50 for each model. For example, the minimal half-lives of the ^12^C emission occur for the parent nuclei ^110^Xe, ^111^Cs, ^112^Ba, ^113^La, and ^114^Ce. Among these minimal half-lives, the half-life with the daughter nucleus ^100^Sn (the parent nucleus ^112^Ba) is shorter than any other minimal half-life. Similar phenomena can also be observed in Figs. 2 and 3. These facts reveal that the CR half-lives are related to the shell effect at *N*_*d*_ = 50, and the shell effect at ^100^Sn is strongest. To explain the shell effect of the CR half-lives shown in Figs. 1–3, the *Q* values of the ^12^C, ^20^Ne, and ^28^Si emissions of these isotopic chains as functions of *N*_*d*_ are shown in Fig. 4. As can be seen from Fig. 4, the shell effect at *N*_*d*_ = 50 is very obvious and the shell effect at ^100^Sn is most pronounced. In the half-life calculations the shell effects are included through the *Q* values. The *Q* value shell effects at *N*_*d*_ = 50 and ^100^Sn lead to the above phenomena. In addition, from Figs. 1–3, it is found that the half-lives by the TM and BKAG formulas become closer and closer to the ones by the ELDM and GLDM with the increase of the emitted cluster mass. This suggests that the TM and BKAG are just suitable for studying heavier cluster emissions.

**Figure 5:**
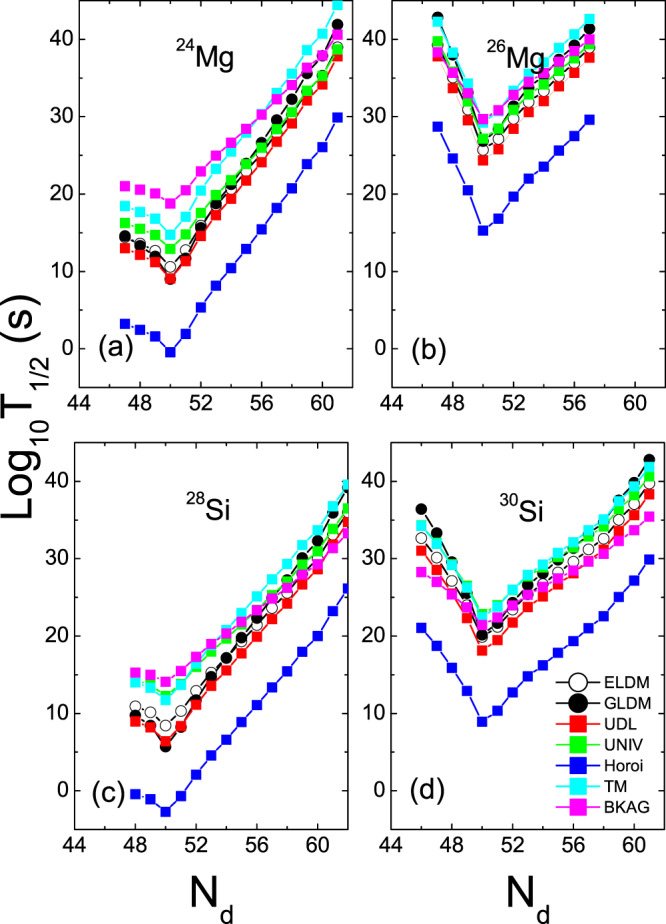
The half-lives of the ^24,26^Mg and ^28,30^Si emissions leading to the daughter nuclei with *Z*_*d*_ = 50 within the ELDM, GLDM, UDL, UNIV, Horoi, TM, and BKAG models (formulas) versus *N*_*d*_.

**Figure 6:**
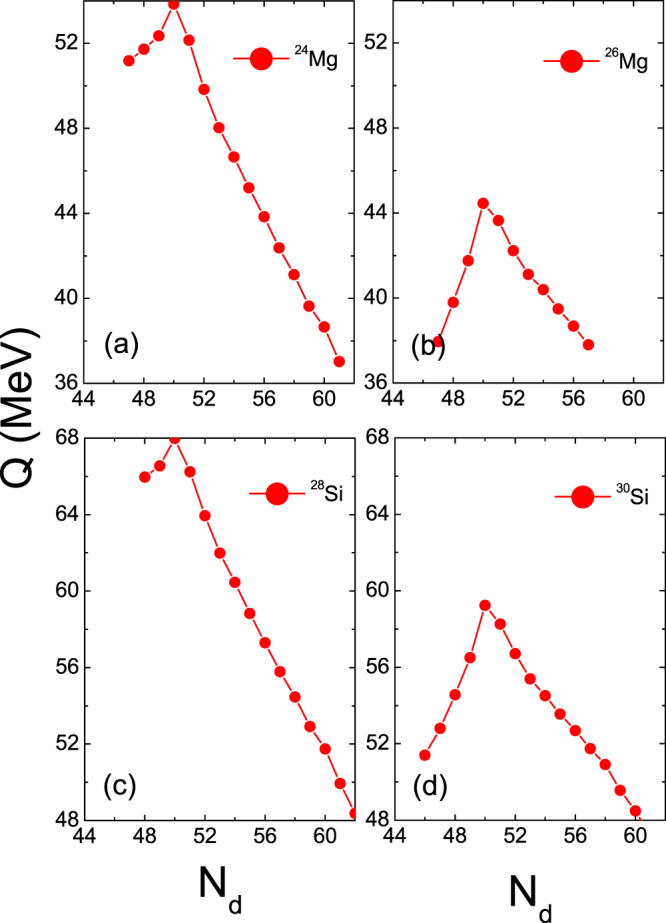
The *Q* values of the ^24,26^Mg and ^28,30^Si emissions leading to the daughter nuclei with *Z*_*d*_ = 50 versus *N*_*d*_.

The clusters ^12^C, ^20^Ne and ^28^Si can be seen as *α*-like ones^76,78^. In addition to the half-lives of the *α*-like CR, the half-lives of the non-*α*-like^78^ (^26^Mg and ^30^Si) CR are calculated as well. For comparing the similarities and differences between the two sorts of cluster emissions, the half-lives of the ^24,26^Mg and ^28,30^Si emissions leading to the daughter nuclei with *Z*_*d*_ = 50 are shown in Fig. 5 as functions of *N*_*d*_, which are calculated with all the models (formulas) except for the NRDX and VSS formulas. From Fig. 5, we can see that for each model the half-lives of the ^26^Mg and ^30^Si emissions are much longer than those of the ^24^Mg and ^28^Si emissions besides the shell effect at ^100^Sn. This implies that the non-*α*-like cluster emissions are more difficult to observe than the *α*-like ones, which is consistent with the conclusion of refs. ^76,78^. In Fig. 6, we plot the *Q* values of the ^24,26^Mg and ^28,30^Si emissions decaying to the *Z*_*d*_ = 50 daughter nuclei versus *N*_*d*_. As can be seen from Fig. 6, the *Q* values of the ^24^Mg (^28^Si) emission are much larger than those of the ^26^Mg (^30^Si) emission in addition to the strong shell effect at ^100^Sn. Small *Q* values of the non-*α*-like cluster decay lead to the long half-lives.

According to the above discussions, one can see that a CR most probably occurs in the decay process where the daughter nucleus has *N*_*d*_ = 50 and its half-life is shortest. Moreover, an *α*-like cluster decay is more probable than a non-*α*-like cluster decay. Therefore, the predicted half-lives of some *α*-like cluster emissions decaying to the daughter nuclei with *N*_*d*_ around 50 based on the ELDM, GLDM, UDL and UNIV models (formulas), which include the ^8^Be, ^12^C, ^16^O, ^20^Ne, ^24^Mg, and ^28^Si emissions, are listed in Table 3. We hope our predictions are useful for searching for new CR in trans-tin region in future experiments. At last, to compare these predictions with those of other models, the half-lives of some clusters within a dinuclear system model (DNSM)^102^ are listed in the last column. Meanwhile, the *Q* values used in the DNSM calculations are given in the penultimate column. By observing Table 3, it is found that the difference is large between our predicted half-lives and those within the DNSM, which is caused by the differences of the *Q* values and models. In other words, the predicted CR half-lives are dependent strongly on the *Q* values and the models. Therefore, it is important to improve the predicted abilities of the nuclear mass models and the approaches of CR by including more reasonable factors of nuclear structure.

**Table 3:** The ^8^Be, ^16^C, ^16^O, ^20^Ne, ^24^Mg, and ^28^Si emission half-lives in the decay processes where the daughter nuclei with *N*_10_*T*_1/2_ around 50 within the ELDM, GLDM, UDL and UNIV models (formulas) are shown in columns 5-8.

Parent nuclei	Daughter nuclei	Emitted clusters	*Q*		log_10_*T*_1/2_ (s)			*Q*^102^	log_10_*T*_1/2_ (s)^102^
			(MeV)	ELDM	GLDM	UDL	UNIV	(MeV)	DNSM
^108^Xe	^100^Sn	^8^Be	10.40	7.20	6.57	6.26	7.15		
^109^Xe	^100^Sn	^8^Be	9.19	11.56	11.09	10.47	11.42		
^110^Xe	^100^Sn	^8^Be	8.07	16.52	16.26	15.20	16.30		
^111^Xe	^100^Sn	^8^Be	7.64	18.74	18.51	17.32	18.48		
^107^Cs	^99^Sb	^8^Be	6.91	24.43	24.65	22.69	24.08		
^108^Cs	^100^Sb	^8^Be	8.64	15.05	14.86	13.82	14.82		
^109^Cs	^101^Sb	^8^Be	10.00	9.54	8.99	8.56	9.42		
^110^Cs	^102^Sb	^8^Be	9.53	11.24	10.77	10.19	11.08		
^111^Cs	^103^Sb	^8^Be	8.58	15.24	14.93	14.02	15.00		
^112^Cs	^104^Sb	^8^Be	7.94	18.29	18.12	16.93	18.01		
^108^Ba	^100^Te	^8^Be	7.46	22.33	22.53	20.75	21.97		
^109^Ba	^101^Te	^8^Be	8.98	14.61	14.41	13.44	14.36		
^110^Ba	^102^Te	^8^Be	10.18	9.86	9.36	8.88	9.69		
^111^Ba	^103^Te	^8^Be	9.65	11.79	11.38	10.76	11.59		
^112^Ba	^104^Te	^8^Be	8.92	14.77	14.50	13.61	14.51		
^108^Xe	^96^Cd	^12^C	14.03	23.03	23.24	21.76	22.79		
^109^Xe	^97^Cd	^12^C	14.53	21.17	21.21	20.00	21.00		
^110^Xe	^98^Cd	^12^C	15.72	17.24	16.87	16.20	17.17		
^111^Xe	^99^Cd	^12^C	15.54	17.74	17.37	16.71	17.66		
^109^Cs	^97^In	^12^C	16.48	16.40	16.19	15.40	16.31		
^110^Cs	^98^In	^12^C	17.63	13.15	12.56	12.26	13.20		
^111^Cs	^99^In	^12^C	18.29	11.43	10.59	10.57	11.53		
^112^Cs	^100^In	^12^C	18.03	12.03	11.22	11.17	12.10		
^113^Cs	^101^In	^12^C	16.97	14.77	14.22	13.88	14.76		
^114^Cs	^102^In	^12^C	16.01	17.54	17.19	16.57	17.41		
^115^Cs	^103^In	^12^C	15.30	19.76	19.54	18.71	19.55		
^109^Ba	^97^Sn	^12^C	17.14	15.85	15.79	14.91	15.75		
^110^Ba	^98^Sn	^12^C	18.63	11.88	11.31	11.05	11.94		
^111^Ba	^99^Sn	^12^C	19.82	9.05	8.10	8.27	9.23		
^112^Ba	^100^Sn	^12^C	21.73	5.07	3.65	4.31	5.45	23.17	0.44
^113^Ba	^101^Sn	^12^C	20.77	6.91	5.67	6.16	7.20		
^114^Ba	^102^Sn	^12^C	18.97	10.84	9.97	10.06	10.94	21.11	4.08
^115^Ba	^103^Sn	^12^C	18.37	12.28	11.51	11.47	12.30		
^116^Ba	^104^Sn	^12^C	17.45	14.62	14.02	13.78	14.56	17.15	16.20
^117^Ba	^105^Sn	^12^C	15.72	19.72	19.53	18.73	19.46		
^109^La	^97^Sb	^12^C	16.77	18.28	18.66	17.28	18.03		
^110^La	^98^Sb	^12^C	18.19	14.27	14.14	13.39	14.17		
^111^La	^99^Sb	^12^C	19.59	10.79	10.15	9.98	10.83		
^112^La	^100^Sb	^12^C	20.72	8.22	7.24	7.46	8.39		
^113^La	^101^Sb	^12^C	21.57	6.41	5.19	5.69	6.69		
^114^La	^102^Sb	^12^C	21.09	7.33	6.18	6.61	7.56		
^115^La	^103^Sb	^12^C	20.30	8.96	7.94	8.22	9.09		
^116^La	^104^Sb	^12^C	19.26	11.28	10.43	10.52	11.30		
^117^La	^105^Sb	^12^C	18.08	14.18	13.62	13.38	14.08		
^118^La	^106^Sb	^12^C	17.11	16.81	16.46	15.95	16.61		
^111^Ce	^99^Te	^12^C	19.04	13.33	13.20	12.49	13.21		
^112^Ce	^100^Te	^12^C	20.36	10.16	9.56	9.40	10.20		
^113^Ce	^101^Te	^12^C	21.41	7.86	6.92	7.14	8.02		
^114^Ce	^102^Te	^12^C	22.26	6.12	4.92	5.41	6.37		
^115^Ce	^103^Te	^12^C	21.70	7.16	6.05	6.46	7.35		
^116^Ce	^104^Te	^12^C	20.62	9.38	8.42	8.66	9.44		
^117^Ce	^105^Te	^12^C	19.89	10.94	10.16	10.23	10.95		
^118^Ce	^106^Te	^12^C	18.57	14.11	13.63	13.36	13.98	17.37	18.20
^119^Ce	^107^Te	^12^C	17.06	18.23	18.12	17.38	17.95		
^112^Ba	^96^Cd	^16^O	25.15	17.81	17.73	16.81	18.07		
^113^Ba	^97^Cd	^16^O	25.90	16.09	15.68	15.13	16.45		
^114^Ba	^98^Cd	^16^O	26.41	14.94	14.30	14.01	15.37	27.98	5.80
^115^Ba	^99^Cd	^16^O	26.07	15.61	14.99	14.67	15.98		
^116^Ba	^100^Cd	^16^O	24.76	18.46	18.14	17.50	18.67	24.65	15.40
^111^La	^95^In	^16^O	25.79	18.03	18.35	17.03	18.21		
^112^La	^96^In	^16^O	26.79	15.81	15.71	14.85	16.12		
^113^La	^97^In	^16^O	27.85	13.60	13.05	12.67	14.05		
^114^La	^98^In	^16^O	28.98	11.39	10.39	10.47	11.99		
^115^La	^99^In	^16^O	29.80	9.86	8.52	8.94	10.56		
^116^La	^100^In	^16^O	29.14	10.97	9.72	10.07	11.59		
^117^La	^101^In	^16^O	27.54	13.96	13.13	13.07	14.37		
^118^La	^102^In	^16^O	26.06	16.98	16.54	16.09	17.21		
^119^La	^103^In	^16^O	24.74	19.94	19.83	19.01	20.00		
^111^Ce	^95^Sn	^16^O	27.00	16.95	17.46	15.99	17.14		
^112^Ce	^96^Sn	^16^O	28.34	14.18	14.11	13.25	14.53		
^113^Ce	^97^Sn	^16^O	29.36	12.18	11.66	11.27	12.66		
^114^Ce	^98^Sn	^16^O	30.51	10.07	9.05	9.15	10.70		
^115^Ce	^99^Sn	^16^O	31.67	8.06	6.57	7.13	8.85		
^116^Ce	^100^Sn	^16^O	33.22	5.57	3.53	4.61	6.57		
^117^Ce	^101^Sn	^16^O	32.12	7.19	5.40	6.28	8.06		
^118^Ce	^102^Sn	^16^O	30.03	10.65	9.39	9.77	11.22	30.55	7.38
^119^Ce	^103^Sn	^16^O	28.23	13.93	13.17	13.08	14.29		
^120^Ce	^104^Sn	^16^O	26.97	16.44	16.00	15.59	16.64		
^121^Ce	^105^Sn	^16^O	25.86	18.81	18.60	17.94	18.87		
^114^Pr	^98^Sb	^16^O	30.35	11.73	11.29	10.83	12.18		
^115^Pr	^99^Sb	^16^O	31.55	9.59	8.62	8.69	10.20		
^116^Pr	^100^Sb	^16^O	32.38	8.16	6.78	7.26	8.89		
^117^Pr	^101^Sb	^16^O	33.05	7.04	5.36	6.13	7.86		
^118^Pr	^102^Sb	^16^O	31.85	8.89	7.49	8.01	9.55		
^119^Pr	^103^Sb	^16^O	30.45	11.21	10.18	10.37	11.70		
^120^Pr	^104^Sb	^16^O	28.98	13.87	13.22	13.04	14.16		
^121^Pr	^105^Sb	^16^O	27.68	16.39	16.04	15.57	16.54		
^122^Pr	^106^Sb	^16^O	26.75	18.32	18.19	17.49	18.34		
^116^Nd	^100^Te	^16^O	32.03	10.05	9.31	9.18	10.58		
^117^Nd	^101^Te	^16^O	32.66	8.95	7.86	8.08	9.56		
^118^Nd	^102^Te	^16^O	33.25	7.95	6.56	7.07	8.64		
^119^Nd	^103^Te	^16^O	32.12	9.69	8.59	8.86	10.25		
^120^Nd	^104^Te	^16^O	30.51	12.42	11.74	11.61	12.77		
^121^Nd	^105^Te	^16^O	29.28	14.66	14.24	13.86	14.85		
^122^Nd	^106^Te	^16^O	28.03	17.09	16.99	16.30	17.14	26.78	17.67
^123^Nd	^107^Te	^16^O	26.75	19.80	20.02	18.98	19.68		
^116^Ce	^96^Cd	^20^Ne	34.21	20.32	20.86	19.19	20.97		
^117^Ce	^97^Cd	^20^Ne	34.82	19.08	19.29	17.97	19.82		
^118^Ce	^98^Cd	^20^Ne	35.03	18.62	18.63	17.52	19.38	34.64	13.04
^119^Ce	^99^Cd	^20^Ne	33.50	21.51	21.91	20.41	22.06		
^120^Ce	^100^Cd	^20^Ne	31.84	24.90	25.73	23.79	25.22		
^121^Ce	^101^Cd	^20^Ne	30.66	27.48	28.58	26.35	27.65		
^115^Pr	^95^In	^20^Ne	35.32	20.08	21.08	18.94	20.67		
^116^Pr	^96^In	^20^Ne	36.02	18.71	19.33	17.59	19.40		
^117^Pr	^97^In	^20^Ne	36.89	17.09	17.25	15.97	17.90		
^118^Pr	^98^In	^20^Ne	37.31	16.29	16.16	15.18	17.16		
^119^Pr	^99^In	^20^Ne	37.52	15.85	15.50	14.75	16.76		
^120^Pr	^100^In	^20^Ne	36.42	17.69	17.60	16.62	18.45		
^121^Pr	^101^In	^20^Ne	34.71	20.80	21.17	19.75	21.32		
^122^Pr	^102^In	^20^Ne	33.27	23.62	24.36	22.57	23.94		
^116^Nd	^96^Sn	^20^Ne	37.58	17.67	18.48	16.55	18.36		
^117^Nd	^97^Sn	^20^Ne	38.18	16.56	17.00	15.45	17.35		
^118^Nd	^98^Sn	^20^Ne	39.07	15.01	14.97	13.90	15.92		
^119^Nd	^99^Sn	^20^Ne	39.66	13.98	13.57	12.87	14.98		
^120^Nd	^100^Sn	^20^Ne	40.68	12.31	11.35	11.19	13.47		
^121^Nd	^101^Sn	^20^Ne	39.08	14.77	14.26	13.68	15.69		
^122^Nd	^102^Sn	^20^Ne	37.05	18.14	18.23	17.11	18.79	37.52	13.30
^123^Nd	^103^Sn	^20^Ne	35.49	20.96	21.45	19.93	21.38		
^119^Pm	^99^Sb	^20^Ne	39.79	15.39	15.67	14.30	16.20		
^120^Pm	^100^Sb	^20^Ne	40.32	14.47	14.36	13.38	15.36		
^121^Pm	^101^Sb	^20^Ne	40.53	14.06	13.72	12.98	14.98		
^122^Pm	^102^Sb	^20^Ne	39.41	15.78	15.71	14.74	16.55		
^123^Pm	^103^Sb	^20^Ne	38.09	17.94	18.18	16.93	18.53		
^121^Sm	^101^Te	^20^Ne	41.08	14.80	14.99	13.72	15.58		
^122^Sm	^102^Te	^20^Ne	41.32	14.35	14.28	13.28	15.17		
^123^Sm	^103^Te	^20^Ne	40.23	16.00	16.18	14.97	16.68		
^124^Sm	^104^Te	^20^Ne	39.11	17.79	18.22	16.79	18.31	37.512	15.51
^125^Sm	^105^Te	^20^Ne	37.77	20.07	20.81	19.09	20.41		
^126^Sm	^106^Te	^20^Ne	36.22	22.88	23.99	21.93	23.02	35.20	18.80
^127^Sm	^107^Te	^20^Ne	34.71	25.85	27.29	24.88	25.78		
^118^Nd	^94^Cd	^24^Mg	45.70	19.10	20.05	17.72	20.61		
^119^Nd	^95^Cd	^24^Mg	46.08	18.43	19.04	17.04	19.99		
^120^Nd	^96^Cd	^24^Mg	46.26	18.05	18.43	16.68	19.66		
^121^Nd	^97^Cd	^24^Mg	46.37	17.81	17.95	16.44	19.42		
^122^Nd	^98^Cd	^24^Mg	46.65	17.29	17.16	15.92	18.96	46.20	14.74
^123^Nd	^99^Cd	^24^Mg	45.34	19.27	19.50	17.95	20.74		
^119^Pm	^95^In	^24^Mg	48.15	17.15	17.92	15.74	18.77		
^120^Pm	^96^In	^24^Mg	48.55	16.48	16.89	15.06	18.15		
^121^Pm	^97^In	^24^Mg	48.97	15.78	15.83	14.36	17.52		
^122^Pm	^98^In	^24^Mg	49.46	14.97	14.65	13.56	16.82		
^123^Pm	^99^In	^24^Mg	49.75	14.48	13.86	13.06	16.37		
^124^Pm	^100^In	^24^Mg	48.82	15.73	15.29	14.36	17.48		
^125^Pm	^101^In	^24^Mg	46.89	18.58	18.72	17.27	20.02		
^121^Sm	^97^Sn	^24^Mg	51.19	14.44	14.58	12.99	16.26		
^122^Sm	^98^Sn	^24^Mg	51.73	13.61	13.32	12.15	15.52		
^123^Sm	^99^Sn	^24^Mg	52.36	12.67	11.92	11.20	14.70		
^124^Sm	^100^Sn	^24^Mg	53.86	10.61	9.01	9.10	12.91	51.97	9.98
^125^Sm	^101^Sn	^24^Mg	52.16	12.76	11.69	11.32	14.77		
^126^Sm	^102^Sn	^24^Mg	49.83	15.92	15.65	14.58	17.57	50.53	11.97
^127^Sm	^103^Sn	^24^Mg	48.03	18.53	18.79	17.25	19.89		
^123^Eu	^99^Sb	^24^Mg	52.80	13.83	13.89	12.38	15.64		
^124^Eu	^100^Sb	^24^Mg	53.48	12.83	12.38	11.37	14.76		
^125^Eu	^101^Sb	^24^Mg	53.98	12.08	11.20	10.61	14.11		
^126^Eu	^102^Sb	^24^Mg	52.80	13.55	12.98	12.14	15.40		
^127^Eu	^103^Sb	^24^Mg	51.21	15.67	15.56	14.32	17.26		
^128^Eu	^104^Sb	^24^Mg	49.71	17.77	18.08	16.48	19.12		
^129^Eu	^105^Sb	^24^Mg	48.24	19.93	20.64	18.70	21.06		
^126^Gd	^102^Te	^24^Mg	54.90	12.51	12.03	11.05	14.40		
^127^Gd	^103^Te	^24^Mg	53.76	13.92	13.72	12.52	15.64		
^128^Gd	^104^Te	^24^Mg	52.56	15.47	15.56	14.12	17.00		
^129^Gd	^105^Te	^24^Mg	51.19	17.34	17.77	16.04	18.65		
^130^Gd	^106^Te	^24^Mg	49.66	19.52	20.37	18.30	20.61		
^121^Sm	^93^Cd	^28^Si	58.56	16.70	17.61	14.94	19.47		
^122^Sm	^94^Cd	^28^Si	59.03	15.99	16.44	14.22	18.84		
^123^Sm	^95^Cd	^28^Si	59.44	15.35	15.40	13.58	18.29		
^124^Sm	^96^Cd	^28^Si	60.11	14.38	13.90	12.60	17.45		
^125^Sm	^97^Cd	^28^Si	60.11	14.29	13.57	12.51	17.37		
^126^Sm	^98^Cd	^28^Si	60.09	14.21	13.31	12.45	17.30	56.66	18.70
^127^Sm	^99^Cd	^28^Si	58.56	16.14	15.71	14.44	18.96		
^128^Sm	^100^Cd	^28^Si	56.77	18.51	18.63	16.90	21.02		
^123^Eu	^95^In	^28^Si	61.82	14.29	14.54	12.47	17.29		
^124^Eu	^96^In	^28^Si	62.37	13.50	13.25	11.67	16.61		
^125^Eu	^97^In	^28^Si	63.08	12.53	11.70	10.68	15.79		
^126^Eu	^98^In	^28^Si	63.52	11.91	10.62	10.04	15.25		
^127^Eu	^99^In	^28^Si	63.53	11.79	10.28	9.94	15.15		
^128^Eu	^100^In	^28^Si	62.41	13.06	11.83	11.27	16.23		
^129^Eu	^101^In	^28^Si	60.53	15.37	14.77	13.65	18.19		
^130^Eu	^102^In	^28^Si	58.77	17.63	17.58	15.99	20.14		
^126^Gd	^98^Sn	^28^Si	65.97	10.90	9.74	8.97	14.32		
^127^Gd	^99^Sn	^28^Si	66.55	10.13	8.44	8.19	13.68		
^128^Gd	^100^Sn	^28^Si	67.99	8.42	5.72	6.41	12.26		
^129^Gd	^101^Sn	^28^Si	66.24	10.29	8.22	8.37	13.80		
^130^Gd	^102^Sn	^28^Si	63.94	12.92	11.73	11.12	16.01		
^131^Gd	^103^Sn	^28^Si	61.99	15.27	14.76	13.56	18.01		
^132^Gd	^104^Sn	^28^Si	60.46	17.17	17.14	15.55	19.66		
^133^Gd	^105^Sn	^28^Si	58.83	19.32	19.79	17.76	21.52		
^128^Tb	^100^Sb	^28^Si	68.11	10.11	8.67	8.14	13.57		
^129^Tb	^101^Sb	^28^Si	68.63	9.43	7.46	7.44	13.01		
^130^Tb	^102^Sb	^28^Si	67.44	10.66	9.03	8.75	14.03		
^131^Tb	^103^Sb	^28^Si	65.72	12.57	11.53	10.75	15.63		
^132^Tb	^104^Sb	^28^Si	64.03	14.54	14.06	12.80	17.30		
^133^Tb	^105^Sb	^28^Si	62.40	16.52	16.54	14.86	19.00		
^134^Tb	^106^Sb	^28^Si	60.95	18.36	18.78	16.76	20.58		
^131^Dy	^103^Te	^28^Si	68.82	10.82	9.56	8.90	14.09		
^132^Dy	^104^Te	^28^Si	67.38	12.39	11.57	10.54	15.39		
^133^Dy	^105^Te	^28^Si	65.78	14.19	13.87	12.44	16.92		
^134^Dy	^106^Te	^28^Si	64.11	16.18	16.36	14.51	18.62		
^135^Dy	^107^Te	^28^Si	62.32	18.43	19.13	16.83	20.55		
^136^Dy	^108^Te	^28^Si	61.24	19.79	20.75	18.25	21.74		

The predicted half-lives of some emitted clusters within the DNSM^101^ are listed in the last column. The *Q* values and half-lives are measured in MeV and seconds, respectively.

## Conclusions

In this article, the CR of the neutron-deficient nuclei in the trans-tin region has been explored within the ELDM, GLDM and several analytic formulas (UDL, UNIV, Horoi, TM, BKAG, NRDX and VSS formulas). Firstly, the ^12^C decay half-life of ^114^Ba has been calculated by all the models. By the comparison between the calculated half-lives and the experimental half-life, it is found that the NRDX and VSS formulas are not so suitable for predicting the CR half-lives in the trans-tin region because the calculated half-lives by the two formulas are less than the experimental lower limit. Next by taking the ^12^C, ^20^Ne, and ^28^Si emissions as examples, their half-lives are predicted by the ELDM, GLDM, and the UDL, UNIV, Horoi, TM, and BKAG formulas. Because the UDL formula originates from the *α*-like *R*-matrix theory and the UNIV formula roots in the fission-like theory, their predicted accuracies are close to the ones by the ELDM and GLDM. However, the half-lives by the ELDM and GLDM are not reproduced with a comparable accuracy by the simple scaling laws (Horoi, TM, and BKAG formulas). With the increase of the emitted cluster mass, only the half-lives by the TM and BKAG formulas become closer and closer to the ones by the ELDM and GLDM. Meanwhile, it is found that the *Q* value shell effects at *N*_*d*_ = 50 and ^100^Sn crucially influence the half-lives, and the daughter nuclei with *N*_*d*_ = 50 have therefore the minimal half-lives. Furthermore, the half-life at ^100^Sn is lower than any other minimal half-life for the same kind cluster emission. It is observed that the half-lives of the non-*α*-like CR decaying to the *Z*_*d*_ = 50 daughter nuclei are much longer than those of the *α*-like CR due to the low *Q* values in the non-*α*-like CR process. At last, the half-lives of some *α*-like cluster emissions, such as the ^8^Be, ^12^C, ^16^O, ^20^Ne, ^24^Mg, and ^28^Si emission half-lives, are predicted by the ELDM, GLDM, UDL, and UNIV models (formulas). We hope these predictions are helpful for future experiments.
